# First report of *Lophomonas* infection in a patient with AML‐2 from Qeshm Island, Persian Gulf, southern Iran

**DOI:** 10.1002/rcr2.906

**Published:** 2022-01-26

**Authors:** Ali Sharifpour, Hossein Zarrinfar, Mahdi Fakhar, Zakaria Zakariaei, Mostafa Soleymani, Elham Sadat Banimostafavi, Maryam Nakhaei

**Affiliations:** ^1^ Toxoplasmosis Research Center, Communicable Diseases Institute, Iranian National Registry Center for Lophomoniasis (INRCL) Mazandaran University of Medical Sciences Sari Iran; ^2^ Pulmonary and Critical Care Division, Imam Khomeini Hospital Mazandaran University of Medical Sciences Sari Iran; ^3^ Allergy Research Center Mashhad University of Medical Sciences Mashhad Iran; ^4^ Toxicology and Forensic Medicine Division, Orthopedic Research Center, Imam Khomeini Hospital Mazandaran University of Medical Sciences Sari Iran; ^5^ Department of Radiology, Imam Khomeini Hospital Mazandaran University of Medical Sciences Sari Iran

**Keywords:** acute myeloid leukaemia, lophomoniasis, Qeshm Island

## Abstract

Immunocompromised patients are frequently more susceptible to pathogens such as protozoa. For the first time, we report a case of pulmonary lophomoniasis in a known case of acute myeloid leukaemia (AML‐2) from Qeshm Island, Persian Gulf, southern Iran. Diagnosis of lophomoniasis was confirmed using microscopy and molecular examinations of bronchoalveolar lavage fluid. She was completely treated with metronidazole (500 mg three times a day for 3 weeks). We conclude that immunocompromised patients with unjustified respiratory disorders should be screened for *Lophomonas* infection.

## Graphical abstract

We report the first case of pulmonary lophomoniasis in a known case of acute myeloid leukaemia (AML‐2) from Qeshm Island, Persian Gulf, southern Iran. Diagnosis of lophomoniasis was confirmed using microscopy and molecular examinations of bronchoalveolar lavage fluid. The patient was treated with metronidazole (500 mg TDS for 3 weeks). We conclude that immunocompromised patients with unjustified respiratory disorders should be screened for *Lophomonas* infection.

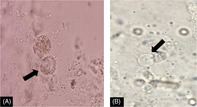

## INTRODUCTION

Lophomoniasis is an emerging human pathogen caused by the multiflagellated protozoan parasite *Lophomonas blattarum*. It lives commensally in the guts of cockroaches and termites. Although the parasite may infect a variety of organs, the lower respiratory tract is the most commonly affected organ. Aspiration of the protozoan cyst into the respiratory tract system is thought to cause infection in humans. The main clinical signs include chronic cough, sputum, low‐grade fever, dyspnoea and haemoptysis. Eosinophilia is diagnosed in about half to one third of patients.^1^

The presence of motile flagellated trophozoites in respiratory secretions, such as sputum and bronchoalveolar lavage (BAL), is used for diagnosis.^1^, ^2^ Currently, microscopic examination, as the gold standard, is frequently used to detect the parasite. Recently, a polymerase chain reaction (PCR) test has been developed to confirm microscopy examination of the mystery protozoan.^2^ Chronic respiratory symptoms with eosinophilia are frequently misdiagnosed as allergies, asthma, Loeffler syndrome and filariasis, and are treated accordingly.^3^ Metronidazole is used as the drug of choice in the treatment of lophomoniasis.^4^

As metronidazole medication is not commonly used in the empiric treatment of patients with chronic respiratory symptoms, an accurate *Lophomonas* diagnosis can be extremely beneficial. At the same time, due to the comparable appearance of ciliated respiratory epithelial cells, there is a risk of overdiagnosis. Lophomoniasis was first reported in humans in China. But so far, most cases have been reported from Iran and a few from other countries (Peru, Spain, Mexico, Malaysia, Turkey and India).^1^, ^5^ There have been several reports of lophomoniasis from several areas in Iran, but there has been no evidence of lophomoniasis in Iran's island regions. Therefore, we here report a case of pulmonary lophomoniasis, for the first time, in a patient with acute myeloid leukaemia (AML‐2) from Qeshm Island, Persian Gulf, southern Iran.

## CASE REPORT

The patient, a 54‐year‐old female from Qeshm Island, Persian Gulf, southern Iran, with a history of shortness of breath, low‐grade fever, chronic productive cough (during the last 6 months) and pleuritic pain, was referred to the emergency ward. She was a known case of AML‐2 undergoing chemotherapy.

**FIGURE 1:**
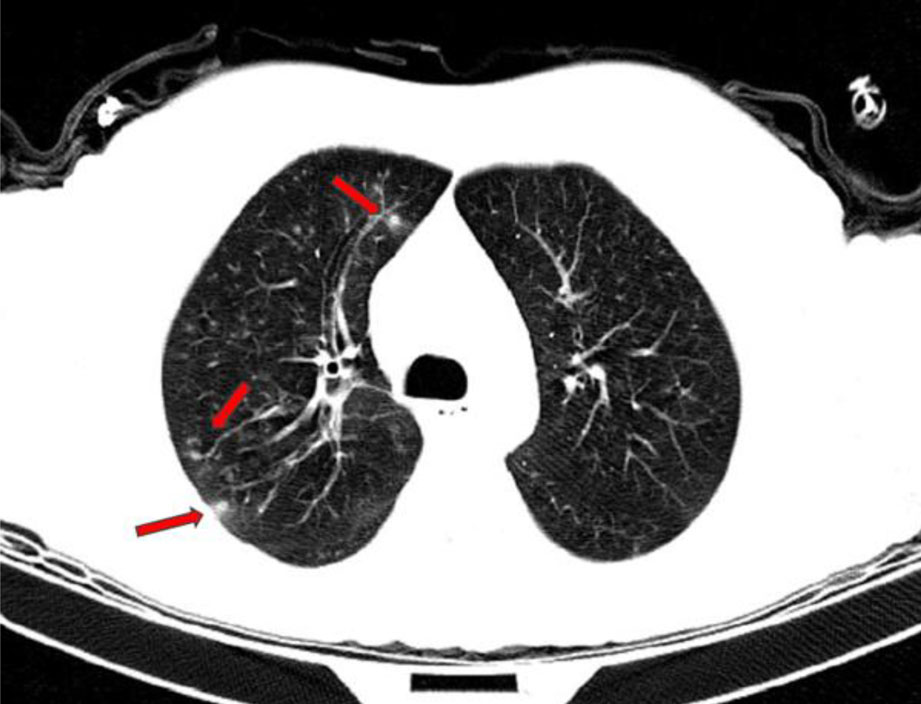
Lung computed tomography scan showing multiple nodules in the right upper lobe of the lung (head of the red arrows)

On examination, the patient's vital signs were as follows: respiratory rate: 18 breaths/min, blood pressure: 110/80 mmHg, heart rate: 90 beats/min, O_2_ saturation (SpO_2_): 93%, temperature: 37.5°C. The patient was admitted to the pulmonologist service. On physical examination, the patient's abdomen was soft and no signs of tenderness or organomegaly were observed in the liver and spleen. A computed tomography scan showed multiple nodules in the right upper lobe of the lung (Figure 1).

The results of laboratory tests were Hb = 7.8 g/dl, white blood cells = 35 ×10^3^/mm^3^, polymorphonuclear (PMN) = 95% and platelets = 39.8 × 10^3^/mm^3^. The patient underwent fibreoptic bronchoscopy due to chronic productive coughs. Two BAL samples were separately subjected to the Iranian National Registry Center for Lophomoniasis (INRCL), Mazandaran University of Medical Sciences, and hospital laboratory to rule out lophomoniasis, tuberculosis (smear and PCR) and possible fungal infection (smear and culture), respectively.

**FIGURE 2:**
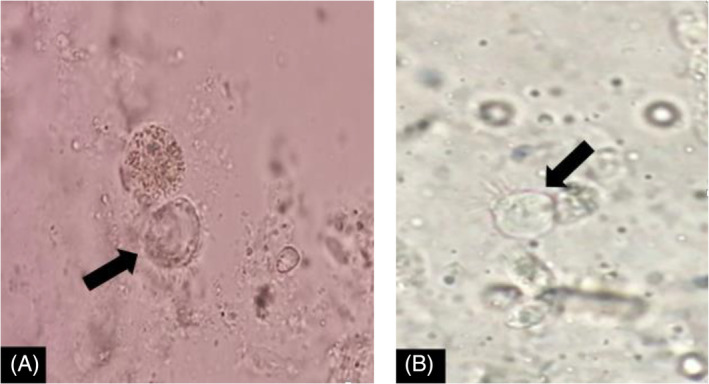
(A, B) Photomicrographs showing trophozoite of *Lophomonas* in a wet smear prepared from bronchoalveolar lavage specimen (black arrows)

The results of mycological tests did not confirm any fungal infection. The BAL sample for *Mycobacterium tuberculosis* was also negative for both smear and PCR. After preparation of a wet smear from a BAL specimen, the live *Lophomonas* trophozoite was identified using a light microscope (Figure 2). The trophozoite was also identified from the BAL sample by a small subunit ribosomal RNA PCR test.^6^ As a result, the *Lophomonas* infection was confirmed. Finally, based on the above‐mentioned laboratory evidence, the patient was treated with oral metronidazole (500 mg/three times a day, for 3 weeks) for lophomoniasis. She was discharged in good general condition from the hospital.

## DISCUSSION

Lophomoniasis is a rare, mysterious and neglected infection that is endemic in different parts of Asia, and most reports have been from Iran and China.^7^ This flagellated protozoan can cause bronchopulmonary lophomoniasis, which is characterized by non‐specific symptoms.^8^ Furthermore, imaging findings in lophomoniasis patients are non‐specific, with condensations being the most common finding.^7^ Affected patients include adults and children who are currently healthy (immunocompetent) or have varying degrees of immunosuppression.^9^ However, we do not have enough information about the pathogenesis of the *Lophomonas* parasite; there is some evidence that it can be an opportunistic infection in patients with any immune system disorder, including immunocompromised and immunodeficient patients. As a whole, based on our experience in the INRCL, any patient with a chronic respiratory disorder, whether immunocompromised, immunodeficient or even immunocompetent, should be screened in terms of lophomoniasis.

Given that the findings of this study confirm the infection of lophomoniasis, it is possible that during the microscopic examination, this parasite, due to its very similarity to the ciliated epithelial cells of the lungs, is misdiagnosed. Therefore, it is recommended to use stained smears for detailed microscopic examination. On the other hand, new advances in molecular identification techniques^6^ have been able to reduce these problems to a great extent, but there are still challenges in identifying lophomoniasis. *Lophomonas* infection has been reported in patients with varying degrees of immunosuppression, such as haematopoietic transplantation^9^ or leukaemia,^10^ and in patients with sinusitis,^11^ asthma,^12^ tuberculosis,^13^ COVID‐19,^14^ invasive pulmonary aspergillosis^15^ and also in the immunocompetent population.^16^ In this case, the clinical features of lophomoniasis in a patient with AML‐2 undergoing chemotherapy are in correlation with previous studies.^10^ However, further clinical studies are needed to make a decision about the management of lophomoniasis associated with leukaemia. Thus, the presented case sheds new light on *Lophomonas* infection among immunocompromised patients.

We conclude that immunosuppressed patients with respiratory disorders must be screened for emerging *Lophomonas* infection. As a whole, we advise pulmonologists, particularly in endemic regions, that in each cancer patient with unjustified respiratory symptoms, lophomoniasis should be considered in the differential diagnosis.

## CONFLICT OF INTEREST

None declared.

## AUTHOR CONTRIBUTION

Hossein Zarrinfar and Maryam Nakhaei were involved in the collection of samples and data. Ali Sharifpour, Mahdi Fakhar, Zakaria Zakariaei and Elham Sadat Banimostafavi were involved in the interpretation, writing and editing of the manuscript. Mahdi Fakhar and Mostafa Soleymani prepared the draft and final version of the manuscript. Mahdi Fakhar and Zakaria Zakariaei judgmentally revised the entire manuscript. All authors reviewed and approved the final version of the manuscript.

## ETHICS STATEMENT

The authors declared that appropriate written informed consent was obtained for the publication of this manuscript and accompanying images.

## Data Availability

The data that support the findings of this study are available from the corresponding author upon reasonable request.
